# Dose-dependent anti-hyperglycemic & anti-dyslipidemic potential of aqueous leaves extract of *Typha elephantina in-vivo* and *in-vitro*

**DOI:** 10.1016/j.sjbs.2023.103868

**Published:** 2023-11-04

**Authors:** Bashir Ahmad, Ali Muhammad Yousafzai, Nasrullah Khan, Ahmed M. Hussein, Amr Kataya, Christian R. Studenik, Mostafa A. Abdel-Maksoud

**Affiliations:** a Department of Zoology, University of Malakand, Chakdara Dir Lower (188000, Khyber Pakhtunkhwa, Pakistan; b Department of Zoology, Islamia College University, Peshawar, Khyber Pakhtunkhwa, Pakistan; c Department of Botany, University of Malakand, Chakdara Dir Lower (188000, Khyber Pakhtunkhwa, Pakistan; d Department of Pharmaceutical Sciences, Division of Pharmacology and Toxicology, University of Vienna, Vienna, Austria; e Department of Biological Sciences, University of Calgary, Calgary, AB, Canada; f Department of Botany and Microbiology, College of Science, King Saud University, P.O. 2455, Riyadh 11451, Saudi Arabia

**Keywords:** Streptozotocin, *Typha elephantina*, Glibenclamide, Antidiabetic activity, Fasting blood glucose (FBG), Oral glucose tolerance

## Abstract

Diabetes mellitus is among the fundamental causes of illness and millions of deaths around the globe are directly attributed to it each year. Current antidiabetic medications often lack sustained glycemic control and carry significant risks of side effects. As a result, the use of plant-based treatments has gained popularity. In this experimental study, we evaluated the aqueous extracts (LQE) of *Typha elephantina* (also known as Elephant grass) leaves collected from freshwater marshes, for their potential anti-hyperglycemic and anti-hyperlipidemic antioxidant effects in healthy streptozotocin caused diabetic-mice. We employed glucose adsorption tests at different glucose levels and glucose diffusion tests to assess the *in-vitro* antidiabetic action of plant extract. For the in-vivo trail, we measured fasting blood glucose (FBG), glucose tolerance (GTT), as well as long-term anti-diabetic, anti-hyperlipidemic, and antioxidant activities. Our results from the glucose diffusion test indicated that the extract was highly effective at both low glucose concentrations (5 mmol L) and high glucose concentrations (100 mmol L). However, the glucose-diffusion ability reached its peaked at an excessively high dosage of the aqueous extract, suggesting a dose-related effect. Similarly, we observed that high doses of TEL.AQ extracts (400 mg/kg body weight) significantly reduced blood glucose levels in healthy mice during the glucose tolerance test (GTT) at 3 h and fasting blood glucose studies (FBG) at 6 h. Furthermore, the high-dose TEL.AQ extract effectively reduced liver-related serum markers and blood-glucose concentration (BGC) in severely chronic diabetic rats. The extract dosage also influenced lipid profile, conjugate and unconjugated bilirubin levels, cholesterol, triglycerides, HDL, and total bilirubin levels. Additionally, after administering a high extract dose, we observed considerable improvement in the liver homogenate markers CAT, POD, and SOD. In contrast, the extract at a low dosage (100 mg/kg), showed minimal, while a moderate dose (200 mg/kg), yielded promising results.

## Graphical abstract

**Figure:**
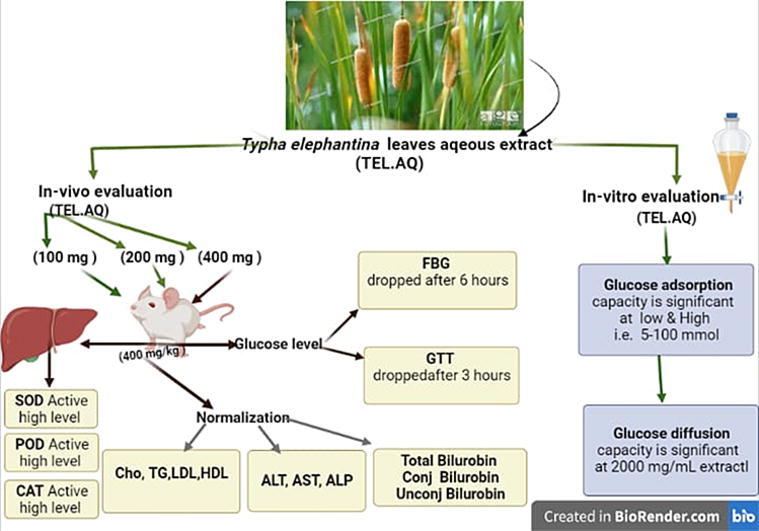

## 1 Introduction

The shortage of insulin, whether partial, complete, or relative, leads to a chronic human condition known as Diabetes mellitus, resulting in hyperglycemia (King, 2012, Gioacchini et al., 2018). Diabetes mellitus manifests in various forms including Type I and Type II diabetes mellitus (Association, 2018), as well as other genetic dysfunction of beta-cell function or insulin action inefficiency (Liston et al., 2017). Condition such as pancreatic pancreatitis or cystic fibrosis, with exocrine origins, are closely related to other endocrinopathies like, acromegaly (Kumaran and Unnikrishnan, 2020, Solis-Herrera et al., 2018, Nogueira et al., 2019). Type I diabetes, an autoimmi = une disease, results from damage to insulin-secreting beta-cells in the Langerhans islets, while Type II diabetes, also known as non-insulin-dependent diabetic mellitus (NIDDM), is characterized by decreased insulin sensitivity in target tissues (Zhou, 2016). Diabetes leads to the generation of high-level free radicals, primarily reactive oxygen species (ROS), through chronic hyperglycemia, originating from glucose auto-oxidation and protein glycosylation in various body tissues (Rahman et al., 2012).These free radicals disrupt cellular metabolism as a by-products, impairing cells function and overall tissue health, impacting defense mechanisms (Mathew et al., 2011). The reduced concentration of antioxidant enzymes and elevated ROS levels contribute to oxidative stress in different tissues, resulting in lipid peroxidation (Matough et al., 2012), cellular necrosis and chronic disorders (Singh et al., 2019). In other words, oxidative stress affects every organ, contributing to diabetes mellitus (DM) (Borg et al., 2019). Studies have shown that individuals with diabetes have a life expectancy around ten years lower than non-diabetics (Tachkov et al., 2020), and the global diabetic population is projected to increase to 360–380 million by 2025–2030 (Wild et al., 2004). These findings emphasized the urgent need for action in addressing the universal issue of diabetes (Zimmet et al., 2014, Wild et al., 2017). Throughout history, humans have turned to medicinal plants with unknown origins for remedies. This search for natural drugs dates back thousands of years (Srivastava, 2018). The usage of plants as herbal remedies has a long history, spanning approximately 5000 years (Pan et al., 2014). In recent times, there has been a growing “Return to Nature” trend, driven by concerns about the side effects of synthetic drugs (Zalavadiya et al., 2013). In previous years, drugs of plant origin have been widely used for both prevention and treatment various ailments (Hosseinzadeh et al., 2015).Medicinal plants are highly regarded globally due to their lack of side effects and rich supply of healthy substances (Sen and Samanta, 2014). In the past decade, medicinal plants have played a therapeutic role in the treatment of diabetes mellitus, which prompting scientific studies to explore further solutions (Yu et al., 2018).

For this study, Hogla, also botanically known as *Typha elephantina* (Roxb) was selected. This plant belongs to the Typhaceae family and possesses a wide range of biological activities, with remarkable phytochemical constituents.. It is commonly found in swamps, channels, shallow waters, and water canals. The Typha genus, as indexed in Kewensis (1895–1958), includes over twenty species. Traditional medicinal practices have associated this plant with various remedies, such as cooling and aphrodisiac effects, as well as the management of leprosy, splenic enlargement, and burning sensations. The plant has been used to treat condition like diarrhea, gonorrhea, and measles, while mature fruits down are utilized as an antiseptic porous absorber for wounds and ulcers. The rhizome of *Typha elephantina* has also been employed in the treatment of various ailments (Rahman et al., 2014, Bulbul et al., 2013; Pand and Thakur, 2014). In the present research, we investigated the antioxidant, anti-hyperlipidemic, and anti-hyperglycemic effects of several *Typha elephantina* Roxb extracts in both healthy and chronic diabetic mice.

## 2 Materials and methods

### 2.1 Preparation of **Typhae elephantina leaf** extract

*Typhae elephantina* leaves were shade-dried, crushed into a powder (550 g), and then mixed with 1000 ml of deionized water. The mixture was agitated for 48 h. Subsequently, the mixture was filtered, and the resulting filtrate was processed using a rotary evaporator under low pressure and high temperature (40 °C) to obtain a thick paste extract (Goorani et al., 2019).

### 2.2 Animals **experimentation**

Male albino mice weighing between 20 and 30 g were used in accordance with the Ethics Committee regulation for Animal Research established by the Department of Biological Sciences (Du Sert et al., 2020). The albino mice were housed in the university's well-equipped animal facility, provided with commercial food, and had ad libitum access to fresh water. Animal Ethical Committee of the Pharmacy Department at Malakand University granted approval for this research with license number (Pharm/EC- (43)/11–03/22).

### 2.3 Chemicals

Streptozotocin and glibenclamide used in this study were obtained from the Biochemistry laboratory, at the University of Malakand.

### 2.4 Acute toxicity test

An aqueous extract of *Typhae elephantina* leaves (TEL.AQ), modified from the method of Kifayatullah et al. (2015) was employed for the toxicity test. Mice were split into four groups of five replicates each after a 14-hour fast. Different concentrations of plant extracts (100, 200, 400, and 800 mg/kg) were administered intraperitoneally to each group using a bulb-tipped steel needle. Subsequently, the mice were provided with unrestricted access to food and water for 48 h while being monitored for symptoms of acute poisoning.

I) In-Vitro Evaluation of Plant Extract for Glucose Absorption Modulation

The study aimed to assess the in-vitro potential of a plant extract in the slowing down glucose absorption from the gastrointestinal tract into the bloodstream, as the rapid postprandial hyperglycemia (PPHG) often masks underlying diabetes state associated with health complications. Several experimental models were used to evaluate the impact of the plant extract on glucose absorption *in vitro*.

**Glucose diffusion test**

Different concentrations of plant extract (100, 200, 500, 1000, 1500, and 2000 mg/mL) were added into 25 ml of a 20 mM glucose solution contained within dialysis bags. Subsequently, the mixture underwent centrifugation in tubes containing 45 ml of 0.15 M NaCl solution at 37 °C while agitating at 150 rpm using an orbit shaker bath. Following the protocols established by Gallagher et al. (2003) and Sidhu et al. (2014), the rate of glucose transfer into the external solution were monitored at 0, 1, 2, 3, 4, and 5 h.. Glucose concentrations in the extracellular fluid were expressed in milligrams per deciliter per hour.

**Glucose adsorption assay**

Following the methodology outlined by Ahmed et al. (2011), 25 ml of a glucose solution (GS) was thoroughly mixed with plant extract samples (at 1 % concentration). The solution was then subjected to agitation while gradually increasing glucose concentrations of 5, 10, 20, 50, and 100 mM. The solution was agitated and allowed to warm in water bath up to 37 °C for six hours. After the incubation period, the solutions were centrifuged at 4,000 g for 20 min to determine the quantity of free glucose present in the supernatant. The procedure for quantifying bound-glucose concentration followed the approach outlined by Ou et al. (2001).

$$Glucose-bound\left(nm\right)\frac{G0-G6}{Weightofthesample(mg)}=Vol.ofthesolution\left(ml\right)$$

Where, G0– glucose levels in mg/dL, at ‘0′ hours and G6 – glucose concentration in mg/dL at ‘6′ hours.

II) *in-vivo* antidiabetic potential
**Normoglycaemic activity**

Before inducing diabetes, we created five groups, each consisting of four animals. Each animal in the group underwent a 6 h fasting period, followed by the administration of a single dose of TEL.AQ extract and glibenclamide i.e.,

Group = A: served as control animals (10 ml saline water)
Group = B: ingest glibenclamide at dose 0.3 mg/kg BW respectively.
Group = C: feed 100 mg/kg BW of (TEL.AQ) extract
Group = D: received (TEL.AQ) extract at 200 mg/ kg BW
Group = E: administered with 400 mg/kg BW of TEL.AQ extract

The level of blood-glucose level for each animal was obtained before and at a 0.5 h, 3 h, and 6 h after extract administration (Patel et al., 2012).

**Oral glucose tolerance test**

The animals were divided into five groups (n = 5), and each group underwent a 14 h fasting period with access to water. The study group received oral administration of TEL.AQ extract following the protocol outlined below:

Control group = reserved normal control animals allowed (10 ml saline water)
Standard group = received glibenclamide (0.3 mg/kg BW).
Extract group 1 = ingest 100 mg/kg BW (TEL.AQ) extract
Extract group II = expose to 200 mg/kg BW of (TEL.AQ) extract
Extract group III = feed 400 mg/kg BW (TEL.AQ) extract

All animals in both groups were given glucose (1 g/kg body weight) 90 min after ingesting the extract plus glibenclamide. At 0, 30, 60, 120, and 180 min following the glucose load, blood glucose levels were measured in each group of animals (Bartoli et al., 2011).

**Anti-hyperglycemic action**

The anti-diabetic effects of the TEL.AQ extract were assessed involving streptozotocin-induced chronic diabetic mice following Wang et al., (2017). In this study, thirty mice were randomly divided into six groups (n = 5) with a baseline glucose level of 220 were selected to participate in a 30-day-long chronic research trail, during which they received daily medication. As a result, BG levels were monitored on Day 0, immediately after treatment initiation, and then again on Days 10 and 30. Additionally, the BW of each animal was recorded on these specified days.

**Group = 1:** These animals were kept as normal control and received only 10 ml of saline water
**Group = 2:** Diabetic control animals, received streptozotocin (50 mg/kg body weight BW following Shiming, et al., 2021.
**Group = 3:** Standard control animals, first exposed to streptozotocin (50 mg/kg) and medicated with glibenclamide (0.3 mg/kg) body weight
**Group = 4:** These mice were earlier exposed to streptozotocin (50 mg/kg) and then feed with TEL.AQ extract (100 mg/kg BW).
**Group = 5:** Animals of this group ingest 50 mg/kg, of streptozotocin and afterward treated with (200 mg/kg BW) of (TEL.AQ) extract
**Group = 6:** Exposed to streptozotocin (50 mg/kg) and then administered with extract of TEL.AQ (400 mg/kg).
**Histopathology**

Histological specimens of liver and pancreas tissues were subjected to staining with Hematoxylin and Eosin (H&E). Subsequently, the prepared slides were scrutinized through a light microscope, specifically the Olympus BX50, and images were captured. The assessment of degeneration in comparison to normal tissue was carried out in accordance with the approach outlined by Almuttairi, 2023. An examination of the structural aspects of the experimental parameters was performed.

### 2.5 Statistical analysis

The results of the present study were analysed using analysis of variance (one-way ANOVA) following Tukey HSD test using the latest version of Graphpad Prism (9.0). Descriptive statistics like means and standard deviations were calculated using MS Excel.

## 3 Results

All the mice remained in good healthy throughout the acute toxicity test, with no symptoms of toxicity, sickness, or mortality observed in any of the groups. Mice were given TEL.AQ extract via acute oral ingestion proved that this plant species is safe even at 800 mg/kg body weight. Consequently, this herb may certainly be used for medicinal purpose at this dosage level. The date shown in Table 1 demonstrates the remarkable glucose-bonding capabilities of various extracts derived from this plant (referred to as TEL.AQ). Our investigations revealed an inverse relationship glucose concentration and the glucose adsorption capacity. Specifically, TEL.AQ extracts displayed effective glucose adsorption at both the low and high glucose levels used in this study (5 and 100 mmol). Notably, the glucose solution at a concentration of 50 mmol displayed significantly higher glucose adsorption (50.86 nm). In contrast, at a concentration of 100 mmol, the value decreased to 39.02 nm.

**Table 1:** Mean glucose bound (nm) and standard deviation (SD) of glucose adsorption ability of (TEL.AQ) extract at 1% concentration.

S.NO	Glucose adsorption easy	
	The concentration of glucose solution (mmol)	Glucose bound (nm)
1	5	3.835 ± 0.1771
2	15	6.205 ± 0.2670
3	25	20.48 ± 2.214
4	35	29.34 ± 1.676
5	75	50.86 ± 5.860
6	100	39.02 ± 0.303

In in-vitro glucose diffusion tests, an extract derived from TEL.AQ considerably reduces the glucose level in the external solution by delaying its membrane diffusion (Table 2). Notably, the extract demonstrated its highest efficacy at a concentration of 2000 g/ml, where after 5 h incubation, the glucose concentration in the external solution was measured at 35.43 mg/dl, compared to 71.98 mg/dl observed in the control group. These findings demonstrate the dose-dependent nature of TEL.AQ extract's impact on glucose diffusion, with more potent effects observed at higher extract concentrations. Furthermore, this research elucidates that even at low glucose levels (5 mmol), the plant extract used in the experiment can effectively bind with glucose molecules, thereby reducing the volume of glucose and impeding its transit through the colon. Consequently, the extracts have shown promise in mitigating postprandial hyperglycemia.

**Table 2:** Mean ± SD of glucose diffusion capacity of (TEL.AQ) extract at various concentrations.

	Glucose concentration in mg/dL				
	0 –Hr.	1 –Hr.	2 –Hr.	3 –Hr.	5 –Hr.
Control (0 mg/ml)	2.13 ± 0.17	26.87 ± 1.08	39.07 ± 0.78	56.43 ± 2.04	71.98 ± 2.27
100	1.93 ± 0.19	23.87 ± 1.05	33.23 ± 0.69	49.96 ± 1.04	58.17 ± 1.12
200	1.85 ± 0.16	24.92 ± 2.67	30.50 ± 0.87	38.81 ± 0.29	50.47 ± 0.82
500	2.13 ± 0.24	21.07 ± 1.24	29.63 ± 0.61	34.96 ± 2.59	49.61 ± 0.75
1000	1.79 ± 0.15	20.16 ± 0.09	25.78 ± 1.09	30.43 ± 0.97	46.27 ± 0.66
1500	2.08 ± 0.36	19.99 ± 0.23	23.50 ± 0.85	27.45 ± 2.02	41.90 ± 0.87
2000	1.99 ± 16.27	16.27 ± 1.25	19.94 ± 0.013	24.16 ± 1.25	35.43 ± 0.78

In normoglycaemic mice, a substantial (P = 0.05) dose-related decrease in fasting blood glucose levels was observed (Table 3). Notably, the administration of 400 mg/kg body weight of the extract effectively lowered glucose levels within 3 h of treatment. The results of the oral glucose tolerance test results revealed that a single dose of (TEL.AQ) extract administered to normal mice led to a substantial decrease in fasting glucose levels, demonstrating its hypoglycemic potential. When assessing oral glucose tolerance in mice, it was found that a high dose of (TEL.AQ) extract (400 mg/kg body weight) yielded reliable results, comparable to those seen in glibenclamide-treated animals. However, the medium and low-dose extracts (100–200 mg/kg body weight) exhibited positive impact on glucose tolerance, although the effects were not statistically significant (Table 4).

**Table 3:** Fasting blood-glucose level (mg/dL) of normoglycaemic mice before and, after treatment.

GroupsGroups	Dose mg/kg	Pretreatment	Post treatment Fasting blood glucose level (mg/dL)			
			Half hour	2 h	4 h	6 h
1	Control 5 ml/kg Saline water	70.8 ± 1.55^a^	70.0 ± 0.04^a^	70.6 ± 0.64^a^	70.3 ± 0.59^a^	70.0 ± 0.08^a^
2	Glibenclamide 0.3	71.0 ± 0.77^a^	72.3 ± 0.3^b^	72.4 ± 0.61^b^	72. 1 ± 0.09^b^	69.9 ± 0.5^b^
3	(TEL.AQ) extract 100	71.0 ± 0.34^a^	75.6 ± 1.2^b^	73.0 ± 0.01^b^	73.1 ± 0.08^b^	72.9 ± 0.6^b^
4	(TEL.AQ) extract 200	71.2 ± 90.6^a^	73.0 ± 0.4^b^	73.5 ± 0.42^b^	72.9 ± 0.45^b^	72.01 ± 0.4^b^
5	Aqueous extract 400	70.6 ± 1.3 ^a^	73.8 ± 0.4^b^	72.0 ± 0.03^b^	72.2 ± 8 0.1^b^	70.03 ± 0.5^a^

Note: Compared to pre-treatment values using Tukey-Test. Superscripted letters ^(a, b, c,)^ shows significant (P < 0.05) difference after comparison to control and among each other.

**Table 4:** Effects of (TEL.AQ) on oral glucose-tolerance in mice (n = 5; mean ± SD).

GroupsGroups	Treatment mg/kg	Blood glucose level (mg/dL)				
		0 min	30 min	60 min	120 min	180 min
1	Control 5 ml/kg saline water	70.2 ± 2.1^a^	72.5 ± 0.6^a^	71.2 ± 0.73^a^	71.2 ± 0.7^a^	70.9 ± 1.2^a^
2	Glibenclamide 0.3	69.7 ± 2.3^a^	88.7 ± 0.9b	81.2 ± 2.20^b^	78.1 ± 1.1^c^	69.7 ± 0.8^a^
3	Aqueous extract 100	70.2 ± 1.0^a^	101 ± 1.2c	91.3 ± 1.5^b^	91.2 ± 0.9^b^	90.5 ± 0.2^b^
4	Aqueous extract 200	70.4 ± 2.5^a^	98.4 ± 0.9c	88.2 ± 1.0^b^	87.5 ± 0.8^b^	80.7 ± 0.6^b^
5	Aqueous extract 400	69.3 ± 0.7^a^	91.8 ± 1.0^b^	85.3 ± 0.4^b^	80.7 ± 0.6^b^	71.1 ± 0.7^a^

Note: Compared to pre-treatment values using Tukey-Test. Superscripted letters ^(a, b, c,)^ shows significant (*P* < 0.05) difference after comparison to control and among each other.

Table 5 displays the impact of different dosages of (TEL.AQ) extract on blood glucose levels in Streptozotocin-induced diabetic mice. The injection of Streptozotocin considerably raises the blood glucose levels in group 2 mice compared to the healthy control mice in group 1 (P < 0.001). Blood sugar levels for all treatment groups were checked on days 10, 20, and 30. The diabetic mice were administered doses of 100 mg/kg, 200 mg/kg, and 400 mg/kg on a monthly basis. The administration of the extract at a dosage of 100 mg/kg did not reduce blood glucose levels in the mice of the treatment group (group 4), even on the last day of treatment, when compared to the control group (group 1). In the case of the 200 mg/kg extract ingestion during the study, no significant impact on outcomes was observed compared to groups 1 and 2. However, animals in group 6 experienced a significant (P < 0.001) reduction in blood glucose levels on day 30 of treatment after receiving a high dose of the extract (400 mg/kg) therapy. Consequently, the effects of the high dosage (group 6) were equivalent to those of glibenclamide, a commonly prescribed anti-diabetic medication, given to the animals in group 3. The extract exhibits substantial anti-diabetic effect when the dosage is increased from 100 to 400 mg/kg body weight.

**Table 5:** Antidiabetic effects of (TEL.AQ) extract on diabetic mice (n = 5; mean ± SD).

Groups	Blood Glucose level (mg/dL)			
	Dose mg/kg body weight	Day10th	Day 14^th^	Day 30th
1	Normal saline 5 ml/kg	71.81 ± 1.30^a^	71.85 ± 0.88^a^	81.10 ± 1.8^a^
2	Streptozotocin 50	246.4 ± 0.95^b^	252.4 ± 2.0^b^	245.2 ± 5.9^b^
3	Glibenclamide 0.3	217 ± 3.20^c^	112.0 ± 1.7^c^	87.3 ± 2.3^a^
4	(TEL.AQ) extract100	245 ± 1.1^b^	222.2 ± 1.4^b^	218.7 ± 1.3^b^
5	(TEL.AQ) extract 200	238.8 ± 3.1^b^	214.5 ± 3.7^b^	140.1 ± 3.8^c^
6	(TEL.AQ) extract 400	231.3 ± 0.7^b^	130.8 ± 2.2^b^	91.5 ± 0.58^a^

Note: Compared all the groups to the normal control group and among each other (Tukey-Test).

Upon comparing our results with those of the normal mice, we observed a significant (P < 0.05) increase in the levels of serum bio-chemicals, including cholesterol, triglycerides, HDL, AST, ALT, and ALP (Group 2) in animals treated with Streptozotocin (50 mg/kg) as detailed in Table 6. The administration of TEL.AQ extract at dose rating 100 and 200 mg/kg body weight (Groups 4 and 5) did not yield any noteworthy effects on serum biochemical marker levels throughout the treatment duration. However, a significant (P < 0.05) standardizing effect was observed at day 30 of treatment in the high-dose rate group (400 mg/kg, Group 6), similar to the levels observed in the group treated with glibenclamide (Group 3).. In contrast to the healthy mice in Group 1, animals given streptozotocin (50 mg/kg) exhibited a substantial (P < 0.05) disturbance in the levels of total bilirubin, conjugated bilirubin, and unconjugated bilirubin. The administration of the extract at dosages ranging from 100 to 200 mg/kg body weight did not substantially normalize the blood biochemical parameters when compared to the diabetic mice (Group 2) in this study. Compared to the groups 1 and 2, ingesting a high-dose extract (400 mg/kg) plus glibenclamide (0.3 ml/kg), a considerable (P < 0.05) reduction in serum biomarker to normal levels was observed.

**Table 6:** Serum parameters of various experimental groups treated with (TEL.AQ) extract and chemicals.

Groups	Treatment mg/kg body weight	Cholesterol mg/dL	Triglycerides mg/dL	HDL mg/dL	AST U/L	ALT U/L	ALP U/L	Total bilirubin	Conjugated bilirubin	Unconjugated bilirubin
1	Normal saline 5 ml/kg	110 ± 1.2	57.2 ± 1.4	41 ± 1.86	40.2 ± 1.5	32.9 ± 1.3	64.3 ± 1.6	0.71 ± 0.01	0.25 ± 0.01	0.51 ± 0.008
2	Streptozotocin 50	110 ± 1.2	78.8 ± 1.1	27.3 ± 1.6	70.6 ± 0.6	64.5 ± 1.5	91.2 ± 1.4	3.2 ± 0.1	2.31 ± 0.02	1.64 ± 0.01
3	Glibenclamide 0.3	113 ± 0.9	57.4 ± 1.6	39.6 ± 3.5	41.2 ± 1.6	33.4 ± 1.4	65.7 ± 1.9	0.8 ± 0.02	0.29 ± 0.01	0.56 ± 0.04
4	(TEL.AQ) extract 100	124 ± 0.8	70.0 ± 1.1	28.2 ± 0.5	66.2 ± 1.4	64.9 ± 1.6	90.8 ± 1.4	2.5 ± 0.33	1.94 ± 0.05	0.23 ± 0.02
5	(TEL.AQ) extract 200	120 ± 1.5	63.2 ± 2.1	33 ± 0.5	59.3 ± 0.5	59.4 ± 1.7	77 ± 4.1	1.9 ± 0.07	0.92 ± 0.05	0.39 ± 0.09
6	(TEL.AQ) extract 400	115 ± 1.8	60.2 ± 0.5	37 ± 1.5	43.3 ± 0.8	35.8 ± 3.6	69.4 ± 0.7	0.83 ± 0.01	0.22 ± 0.007	0.55 ± 0.02

Note: Compared all the groups to the normal control group and among each other (Tukey-Test).

The levels of liver catalases and superoxide dismutase in each experimental group are summarized in Table 7. It was observed that animals in group 2, which were subjected to streptozotocin (50 mg/kg) exhibited significantly lower levels of the liver (CAT), peroxidases (POD), and superoxide dismutase (SOD) when compared to control mice in group 1 (P < 0.05). Notably, CAT, POD, and SOD activities in diabetic mice given TEL.AQ extract at a dosage of 100 mg/kg (Group 4) did not show any improvement, yielding identical outcomes to those observed in Group 2. Conversely, the administration of the TEL.AQ extract to group 5 at a dosage rate of 200 mg/kg had a positive impact on these parameters. However it was determined that this effect was statistically insignificant. Significantly, there was a noteworthy (P < 0.05) increase in liver CAT, POD, and SOD levels in mice given 400 mg/kg of body weight in group 6. As indicated in Table 7, feeding the animals with glibenclamide at a rate of 0.3 ml per kilogram of body weight had a substantial (P < 0.05) regularizing impact on liver antioxidant indicators, with the results in animals from group 6 being nearly identical to those in group 3. Furthermore, measurements of body weight on different days revealed significant changes in body mass, confirming the beneficial effects of the TEL.AQ extract (Table 8).

**Table 7:** Antioxidants markers of liver tissue homogenate of various experimental groups treated with various doses of (TEL.AQ) extract and chemicals.

Groups	Treatment mg/kg body weight	CAT (U/mg protein)	POD (U/mg protein)	SOD(U/mg protein)
1	Normal control normal saline 5 ml/kg	7.36 ± 0.09^a^	11.4 ± 0.61^a^	26.81 ± 1.31^a^
2	Diabetic control Streptozotocin 50	2.36 ± 0.22^b^	6.1 ± 0.26^b^	10.63 ± 0.96^b^
3	Glibenclamide 0.3	7.0 ± 0.08^a^	11.1 ± 0.2^a^	24.84 ± 0.96^a^
4	(TEL.AQ) extract 100	3.5 ± 0.09^b^	6.3 ± 0.04^b^	14.41 ±.96^c^
5	(TEL.AQ) extract 200	4.8 ± 0.17^c^	8.7 ± 0.20^c^	18.17 ± 1.0^c^
6	(TEL.AQ) extract 400	6.9 ± 0.35^a^	10.9 ± 0.12^a^	23.7 ± 2.1^a^

Note: Compared all the groups to normal control group and among each other (Tukey-Test).

**Table 8:** Changes in body weight of mice during treatment (n = 6).

Groups	Treatment mg/kg body weight	Average body weight in days (mg) as mean ± SD			
		Day 1st	Day 10th	Day 20th	Day 30th
1	Normal saline 5 ml/kg	24.25 ± 1.75^a^	27.63 ± 2.2^a^	25.21 ± 1.3^a^	26.65 ± 0.59^a^
2	Streptozotocin 50	27.65 ± 0.46^a^	22.97 ± 0.5^b^	21.45 ± 0.9^b^	19.28 ± 0.7^b^
3	Glibenclamide 0.3	28.74 ± 1.19^a^	23.89 ± 0.9^a^	23.20 ± 0.5^a^	26.10 ± 0.7^c^
4	A(TEL.AQ) extract 100	27.33 ± 2.00^a^	23.05 ± 0.7^a^	22.06 ± 0.6^b^	22.29 ± 0.38^b^
5	(TEL.AQ) extract 200	26.35 ± 2.17^a^	23.44 ± 0.1^a^	22.63 ± 1.2^b^	23.12 ± 1.6^a^
6	(TEL.AQ) extract 400	27.90 ± 1.6^c^	23.95 ± 0.5^a^	22.98 ± 0.9^b^	24.82 ± 0.3^a^

Note: Compared all the groups to normal control group and among each other and Superscripted letters ^(a, b, c,)^ shows significant (P < 0.05) difference after comparison to control and among each other (Tukey-Test).

### 3.1 Histopasthology

**Fig. 1:**
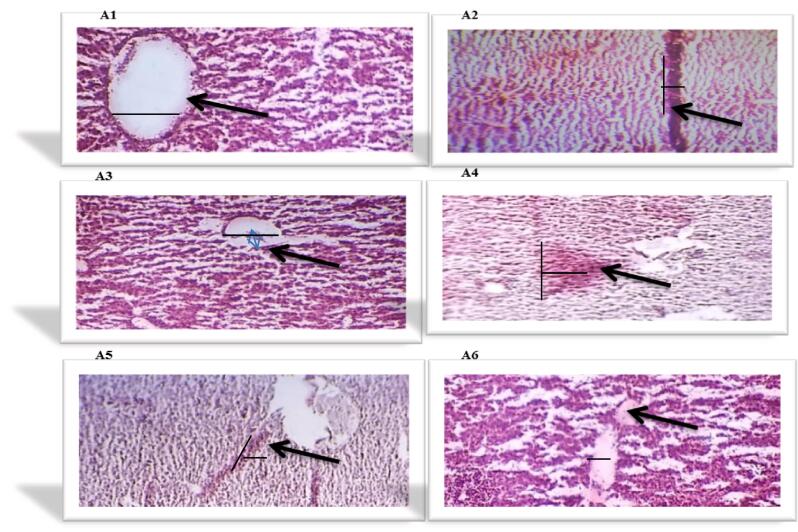
Showing liver histological structures of various experimental animal groups i.e A1, A2, A3, A4, A5 and A6 respectively.

**Fig. 2:**
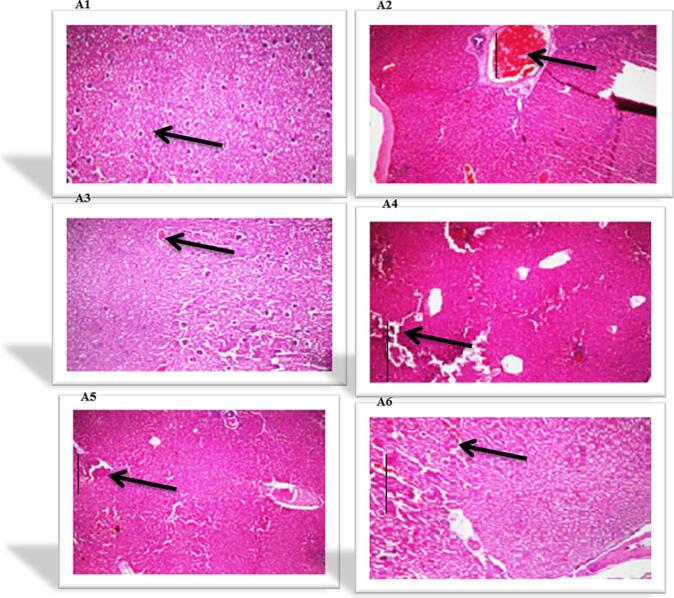
Showing pancreas histological images of different experimental animal groups i.e A1, A2, A3, A4, A5 and A6.

All liver and pancreas histological structures **(A1, to A6)** are shown in Fig. 1and Fig. 2 as:

**A1**: (group = 1): showed that liver parenchyma linings of endothelia of central veins had normal shape, and normal hepatic portal vein and artery while the pancreatic architecture with intact exocrine tissue and islet cells surrounded by delicate capsule.

**A2**: **(**group = 2) ingest streptozotocin (50 mg/kg BW), revealed damage to liver parenchyma. Swelling, necrosis of hepatocytes, deterioration, and minor steatosis are seen. Similarly pancreas with exocrine tissue showing massive degeneration, congestion and obstructions of pancreatic cells thus shows hyperglycemia.

**A3**: (group = 3) feed glibenclamide 0.3 ml/kg BW (standard antidiabetic drug) showed highly significant remedial outcome on liver parenchyma and liver cells seen normal with the background. Negligible necrosis is seen.The pancreatic tissues display significant regeneration and improvement in their histoarchitecture equivalent to normal control animal group.

**A4**: **(**group = 4**)** received **(**TEL.AQ) extract at a 100 mg/kg dose rate of BW, hepatocytes show no important effects on liver parenchyma. Inflammation, necrosis, swelling, degeneration, and steatosis are seen. In the same way the pancreas display sever atrophy and occlusion of the cell histology.

**A5**: (group = 5) ingest (TEL.AQ) extract at a 200 mg/kg dose rate of BW, no substantial influence on liver and on pancreas tissues. Inflammation, necrosis, swelling, degeneration, and mild steatosis were observed.

**A6**: = 6 feed (TEL.AQ) extract at a 400 mg/kg dosage rate of BW), the liver and pancreas shows integral parenchyma tissues and hepatocytes with normal islets of Langerhans and the backdrop of minor inflammation.

## 4 Discussion

To induce diabetes in mice, streptozotocin (STZ) was employed, a commonly used medication in various experimental models for diabetes induction. This medication destroys the beta cells that produce insulin. The result is reduced endogenous insulin release and tissue glucose uptake (Zhang et al., 2012). This experimental study demonstrated that STZ-induced diabetes led to a significant decrease in blood insulin levels and a notable increase in serum glucose levels. Different biochemical parameters of serum get elevated by STZ that include ALT, AST, ALP, total bilirubin along with conjugated and unconjugated bilirubin plus lipid profile and antioxidants of the liver like SOD, POD, and CAT. STZ-induced diabetes was also confirmed from and following research groups (Okoduwa et al., 2017, Alimohammadi et al., 2013), where they proved that STZ caused hyperglycemia in rats.

Diabetes mellitus is a metabolic disease associated with the abnormal or faulty digestion of carbohydrates (Giacco et al., 2016), fat (Hetz, 2012), and protein (Gomez-Serrano et al., 2017) metabolism, leading to either disrupted glucose homeostasis or impaired insulin secretion (Sudha, 2019).

This disease is linked to an increase of complications such as coronary heart disease (Gaggini et al., 2013), stroke (Susan van et al., 2010), liver (Zoppini et al., 2014), and kidney damage (Tuttle et al., 2014). In modern days of sophisticated technology, more medicines are available in the market but they are always associated with the risk of harmful side effects in the short as well as a long run and cannot provide lasting positive effects with control over diabetes especially (Prabhakar and Doble, 2011). Consequently, herbal medicine is gaining popularity due to its efficiency, safety, affordability, acceptability, and fewer associated side effects (Khan and Ahmad, 2019). This experimental study was therefore aimed to investigate the *in-vitro* and *in-vivo* antidiabetic and hypolipidemic potential of TEL.AQ extract in STZ-diabetic mice, comparing it with glibenclamide, a standard antidiabetic drug.

Glucose absorption test and glucose diffusion assays were performed to study the *in-vitro* antidiabetic effect of the plant under experiment. The glucose adsorption capability of **(**TEL.AQ) extract exhibited maximum glucose bound at 50 mM of glucose solution (50 nm). Available data proves that the quantity of the plant’s ingredients is directly proportional to the ability to inhibit glucose absorption such as soluble polysaccharides, described by Barapatre et al., 2015, Gallagher et al., 2003 and Sidhu et al., (2014). Postprandial hyperglycemia is efficiently reduced by this extract.

The (TEL.AQ) extract also substantially reduced glucose diffusion compared to control values, consistent with previously published study (Misbah et al., 2013), which looked at the fruit, extracts, and fractions of *Ficus deltoidea* and found that they exhibit substantial anti-diabetic and antioxidant properties. In-vitro glucose diffusion is useful for determining the impact of plant fibers on the GI tract's ability to absorb glucose more slowly. Along with glucose adsorption, the placement of glucose within the network of fibers and the physical barrier supplied by the fiber components to glucose molecules affect how quickly glucose diffuses. The most recent results align with fractions high in insoluble fiber isolated from *Averrhoa carambola* (Chau et al., 2004).

Given the positive *in-vitro* results, the TEL.AQ extract was selected for *in-vivo* studies. During the first three hours of treatment, the extract administration at various doses did not significantly reduce glucose concentrations in fasted mice. However, a dose-dependent hypoglycemic effect was observed after six hours, with high-dose extract treatment exhibiting significance (P < 0.001). Glibenclamide administration stimulated insulin release from pancreatic beta cells, leading to significant glucose reduction, consistent with previous findings (Adika et al., 2012, Alinejad-Mofrad et al., 2015). Generally, the oral glucose tolerance test (OGTT), measures the ability of body in digesting glucose, which is the fundamental source of body energy, or clears it out of the bloodstream (Brunner and Suddarth, 2010). In normal mice, the findings of an oral glucose tolerance test demonstrated that low to medium dosages of extract exhibit minimal efficiency. However, there in the case of high-dose extract plus glibenclamide, higher glucose tolerance was seen at 3 h. Khalid et al. (2011) and Chen et al. (2018) also presented similar information. After reporting on the anti-diabetic and hypolipidemic efficacy of anthocyanin extract from black soybean seed and coat in streptozotocin-induced diabetic mice, the researchers then examined the aqueous extract of Ipomoea carnea leaves in diabetic rats.

In the present work, the importance of extract for all doses during the first twenty days of treatment and blood glucose level was not reduced significantly. However, upon carrying out the analysis on the 30th day, high extract doses plus glibenclamide significantly reduced the blood-glucose level. Apparently, this is owing to the improvement of insulin action and its positive effect on glycemic control in the peripheral tissues. Therefore, the beta cell function of the pancreas received a positive effect, and/or the beta cell mass was decreased along with the induction of lytic changes in pancreas islets by STZ as observed in the histological analysis. This study significantly reveals that the decrease in blood glucose was both time and dose-dependent upon the dual treatment. Our results aligns also with the previously carried out (Shewasinad et al., (2018) investigation which studied the antidiabetic effects of *Thymus schimperi ronniger* leaves methanol extract and fractions in normal and streptozotocin induce diabetic mice. Moreover, it was reported (Kim and Kim, 2012), similar results in streptozotocin-induced diabetic mice exploring insulin trophic and hypolipidemic action of *Ecklonia cava*.

On the other hand, rise in the levels of blood-glucose in STZ-brought diabetes is frequently accompanied by a rise in serum cholesterol, triglycerides, and a drop in HDL values (Jeong et al., 2010). The hyperglycemia was accompanied by a dyslipidemic disturbance, as evidenced by the considerable increase in Cholesterol, Triglycerides, and HDL. The diabetic group in this study exhibited significant increase in cholesterol, triglycerides, and HDL levels following the research of Movahedian et al. (2010), which reported anti-hyperlipidemic action of *peucedanum pastinacifolium extract* in streptozotocin-induced diabetic-rats. When diabetic mice were given (TEL.AQ) extract, their blood glucose levels dropped and their lipid metabolism improved, resulting in a substantial reduction in serum cholesterol, triglycerides, and an increase in HDL-c levels when compared to diabetic rats. The use of high-dose extract and glibenclamide on the final day of treatment improved the lipid profile approaching normal values. These results are also comparable with published research (Haidari et al., 2012), which investigated the effect of green tea extract on lipid profile, serum glucose, and body weight in streptozotocin-induced diabetic rats.

Aside from its anti-hyperlipidemic and anti-diabetic properties, the (TEL.AQ) extract also protects against hepatic dysfunction. One of the best-known liver function tests includes increased levels of AST, ALT, and ALP (Robles-Diaz et al., 2014), as well as bilirubin, a metabolic result of the breakdown of heme (Teschke et al., 2017). Bilirubin levels rise when hepatocytes are injured, biliary excretion into the duodenum is blocked, and hepatic uptake is lost (Ramaiah, 2007). In the present study, STZ-induced diabetes in animals exhibited a substantial upsurge in the level of AST, ALT, ALP, total bilirubin, conjugated bilirubin, and unconjugated bilirubin as compared to control groups. Administration of extract (400 mg/kg/body weight) and glibenclamide has significantly lowered the above reference serum biomarkers as compared to diabetic mice. The ameliorative effect of **(**TEL.AQ) extract was confirmed through histological findings in liver tissues, by reducing liver cirrhosis. Comparable findings were demonstrated by published report (Narsimhulu et al., 2019), which studied the liver-protective and renal-protective activity using methanolic extract of Cleome viscosa.

STZ's diabetogenic effect, supported by pancreatic beta cell loss, resulted in oxidative stress due to a reduction in the antioxidant scavenger system, resulting in lower levels of CAT, POD, and SOD, which is a clear indicator of *in-vivo* free radical formation and tissue impairment (Yang et al., 2011, Kaleem et al., 2006). In comparison to diabetic mice, treatment with (TEL.AQ) extract (400 mg/kg BW) resulted in a significant increase in antioxidant enzymatic and non-enzymatic status (SOD, CAT, and POD levels), respectively. These findings matched those of Ghatak and Panchal, (2012), which described oryzanol's anti-diabetic properties and their link to antioxidant status. The significant increase in antioxidant indices in diabetic animals' liver tissue suggests that the (TEL.AQ) extract effectively boosted antioxidant potential *in-vivo* by serving as a powerful superoxide radical and singlet oxygen quencher. This conclusion is consistent with what has been found by other researchers (Son et al., 2011). Furthermore, the current work, which demonstrates the *in-vivo* antidiabetic efficacy of (TEL.AQ) extract in normal and diabetic mice, is the first of its kind, and it was used to validate the *in-vitro* findings of the extract's participation in carbohydrate metabolism. The current study's findings, however, clearly show that the (TEL.AQ) extract might be developed as an anti-diabetic medication with a considerable influence on lowering high blood glucose levels.

## 5 Conclusions

From this research study, it was concluded that extract of *Typha elephantina* leave has high potential for treating diabetes and oxidative damage. It effectively regulated blood-glucose levels and improved various serum biomarkers and lipid parameters. The extract also exhibited antioxidant properties by enhancing tissue POD, SOD, and catalases CAT (cellular antioxidants) levels. On the basis of the above solid grounds, this extract can be used for the treatment of many liver disorders. Extract from this plant may also be employed for fatal and chronic diseases. Overall, we supplied strong evidence that TEL.AQ extract possesses antidiabetic and antioxidant properties, and has the potentiality to be used as the best source of advanced medication. Thus, we also suggest further exploration of this valuable plant for this sort of detailed study.

## Funding

No external funding.

Consent Statement: Not applicable.

## Availability of data

Not applicable.

## Declaration of competing interest

The authors declare that they have no known competing financial interests or personal relationships that could have appeared to influence the work reported in this paper.
